# Challenges in the Rapid and Responsible Integration of Generative Artificial Intelligence (AI) Into a New Medical School Curriculum

**DOI:** 10.7759/cureus.86796

**Published:** 2025-06-26

**Authors:** Gabrielle Rueff, Paul Monaco, Antonio E Rusinol, Mark Hernandez

**Affiliations:** 1 Medical Education, East Tennessee State University Quillen College of Medicine, Johnson City, USA

**Keywords:** curriculum, generative artificial intelligence, integration, medical education, problem-based learning (pbl)

## Abstract

This study describes a systematic approach to integrate generative artificial intelligence (AI) into our new medical education curriculum at the Quillen College of Medicine while maintaining academic integrity. As medical schools navigate the widespread adoption of large language models, we implemented a five-step process to address the policy recommendations of the Association of American Medical Colleges (AAMC) on AI integration. First, surveys assessed AI usage patterns among students, revealing increasing adoption (from 24% to 77%) between May 2024 and February 2025. Second, clear professionalism guidelines were established, prohibiting AI use in generating learning objectives, writing Subjective, Objective, Assessment, and Plan (SOAP) notes, or completing assignments while permitting its use in research applications or otherwise in specific course settings when given permission to do so by faculty. Third, an institutional grant ensured equitable access to AI platforms for all students entering in Fall 2024. Fourth, AI was integrated into controlled educational settings, particularly within problem-based learning (PBL) for first-year students and team-based learning (TBL) for second-year students, with structured evaluation criteria. Finally, specialized training on ethical AI use was provided to students transitioning to clinical clerkships. Survey data indicated that students found AI exposure beneficial (91% agreement) and helpful for researching learning objectives (94% agreement), though confidence in AI's accuracy was lower (85% agreement). Students prioritized summarizing learning materials and testing understanding as important AI applications while valuing the ability to function as clinicians both with and without AI. Our approach demonstrates a balanced integration strategy that encourages responsible AI adoption while maintaining educational integrity in medical training.

## Introduction

The Quillen College of Medicine (QCOM) at East Tennessee State University (ETSU) adopted a new curriculum in Fall 2023 called TRAILS (Team-based Rural Applied Integrated Learning System). This curriculum integrates basic and clinical sciences while providing early clinical experiences and emphasizing interprofessional training and community outreach.

Most medical schools are at the early stages of adopting generative artificial intelligence (AI) [1], and like many medical education programs across the United States, QCOM has been challenged to rapidly incorporate training and exposure to AI [2] while maintaining appropriate professionalism policies. The widespread use of large language models (LLMs) such as ChatGPT among our students raised several questions about how to best incorporate these tools into medical education without breaching existing professionalism standards.

This challenge was compounded by the absence of clear national guidelines relating to the use of AI in medical educational settings and the fact that many of our faculty may be no more familiar with AI than our students. The updated Foundational Competencies for Undergraduate Medical Education, released in December of 2024 [3], recommend using appropriate resources, including emerging technologies, under competencies for medical knowledge and systems-based practice, although AI is not specifically mentioned. The Association of American Medical Colleges (AAMC) also recommends the responsible integration of AI, including ethical considerations, equitable access, and continuous learning and adaptation. However, there is limited data on how to best implement these recommendations [4].

We present here our approach to rapidly adapting and addressing the AAMC policy recommendations. Our aim was to create an educational setting that facilitates the integration of AI technology not only by educators but also by learners in the classroom while preparing our preclinical students for the challenges of AI adoption during their clerkship training.

## Materials and methods

Surveying students on AI usage

In Spring 2024, we developed and distributed a satisfaction instrument, which is available at the AAMC Advancing AI Resource Collection (see Appendices [5]), to gather information about current usage and viewpoints regarding AI technology in medical education. The survey was administered when our second cohort of students in TRAILS completed their first year, and it was administered to incoming students in our third cohort in the new curriculum in Fall 2024.

The survey included items rated on a 4-point Likert scale (1=not at all important to 4=very important) to measure the relative importance of various AI uses. An open-ended question allowed participants to express their thoughts about integrating AI into their education. The survey was distributed through Qualtrics to students enrolled in our pre-clerkship program as part of an East Tennessee State University (ETSU) Institutional Review Board (IRB)-approved research study (approval number: 0524.7e-ETSU; date: May 9, 2024). Faculty were also invited to participate in the survey.

Instituting a professionalism policy on AI use

In Fall 2024, entering students in our third cohort received guidelines on AI use as part of our problem-based learning (PBL) component in TRAILS. The guidelines established clear boundaries such that (1) students were prohibited from entering any specific information about PBL cases, case stories, or patient information into any AI chatbot, (2) students could not use AI to develop learning objectives (LOs) for PBL cases or to write Subjective, Objective, Assessment, and Plan (SOAP) notes, (3) students were permitted to use AI for research after they had developed their LOs, and (4) unless explicitly permitted by faculty or course directors, students could not use AI for any submitted assignments, either individual or group, and they could not use it for examinations of any type.

Implicit, based on our honor code, suspected violations would be immediately reported to the Honor Council, and students found to have violated the guidelines would be referred to appropriate disciplinary processes. There were no indications that students did not adhere to the guidelines described above.

Providing access to AI applications in a controlled setting

We developed and received an institutional grant to make paid versions of AI applications accessible in a controlled educational setting for all entering medical students in Fall 2024. This grant provided an opportunity for students to use AI applications in selected PBL sessions.

We identified PBL in the M1 year as the most appropriate place to introduce AI applications in a controlled setting. The grant funds supported access fees for all medical students (a maximum of $20 per month for three months) who would otherwise be unable to afford premium AI services. This ensured equitable access during the semester, where students would be instructed to use AI in selected PBL cases.

Introducing AI applications during selected curriculum activities

In Fall 2024, second-year medical students (M2s in our second cohort of students in TRAILS) and first-year medical students (M1s in our third cohort in TRAILS) were given permission to use AI during PBL (M1s) and during selected team-based learning (TBL) or TBL sessions (M2). In these controlled settings, students could check and review information provided by LLMs.

These hands-on experiences provided meaningful feedback on the integration process and helped build shared best practices between faculty and students. For example, in our PBL sessions, students typically generate LOs for independent study as they work through a clinical case. They then come back in a second session to review what they learned, to teach each other, and to compare their LOs to faculty-generated objectives. We allowed M1 students to use AI while researching and critically assessing information for their LOs, but they could not use AI to develop their LOs. They were asked to compare and contrast findings related to their LOs using AI and traditional research methods using the criteria in Table 1. 

**Table 1:** Qualitative criteria to assess value of information in PBL and TBL settings PBL: problem-based learning; TBL: team-based learning

Criterion	Description
Relevance	Does the output directly address the query?
Accuracy	Is the information provided correct based on cited reliable data or sources?
Clarity	How easy is it to understand the output? Is the language clear and straightforward?
Completeness	Does the output address all aspects of the query? Are there missing details that are critical to a full understanding of the response?
Conciseness	Is the information provided in a concise manner without unnecessary detail?
Appropriateness	Is the tone/style of the output appropriate for the intended audience, and is the level of detail suitable for preclinical medical students?

During selected TBL sessions, M2 students were provided prompts and instructed to compare their initial results from matching case presentations to various attributes (treatments, drugs, adverse effects) with those provided by chatbots. These students also evaluated their findings based on the criteria outlined in Table 1. Each group was required to submit the transcript of their findings, including the prompt they used and the specific LLM (ChatGPT, Gemini, Copilot, etc.). 

Emphasizing ethics of AI and safe practices during the transition to clerkships

As AI transforms healthcare, we recognized it was crucial for our students starting clerkships to understand safe AI usage practices. During the week prior to clerkship rotations, rising third-year medical students (M3) attended a session on AI that covered the following: tips for safe practices in clinical settings, navigating different hospital policies during electives, prioritizing patient privacy and data security, ensuring compliance with regulations, and consulting faculty/supervisors to verify AI-generated insights.

We presented survey data showing how students had been using AI in our classrooms while emphasizing the need for caution in clinical settings. Students were advised to adhere to each institution's guidelines, which may vary across clinical sites. This is particularly important since our M3 are exposed to clinical settings in a Veterans Affairs (VA) hospital and/or in one of several community hospitals in a regional healthcare system. Additionally, many of our students will engage in away rotations, a common practice in US medical schools, in their senior year.

## Results

**Figure 1:**
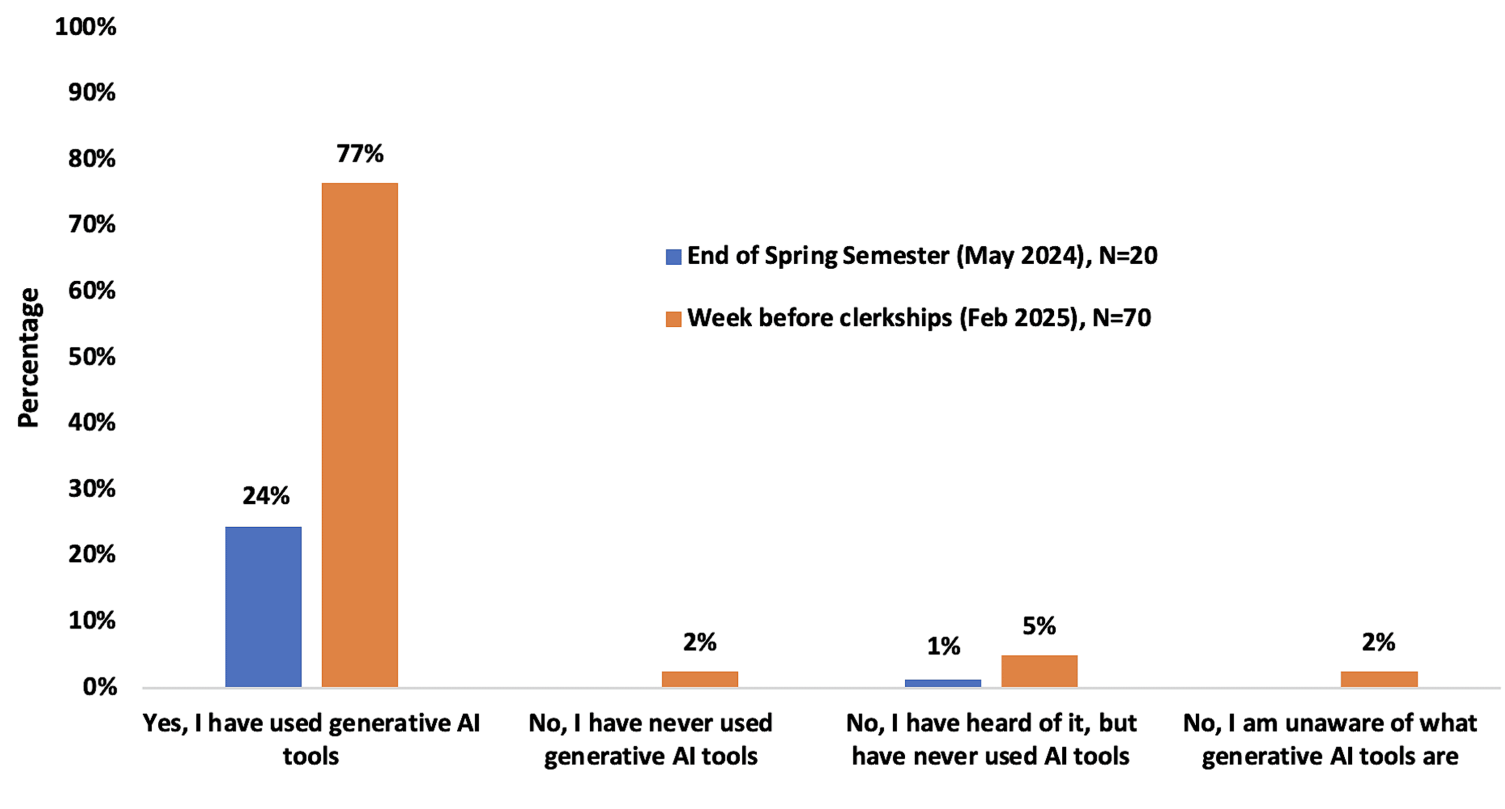
Frequency of AI utilization by our second cohort of students (M2s) AI: artificial intelligence

Use of AI for learning purposes increased between May 2024 and February 2025 (Figure 1). 

**Figure 2:**
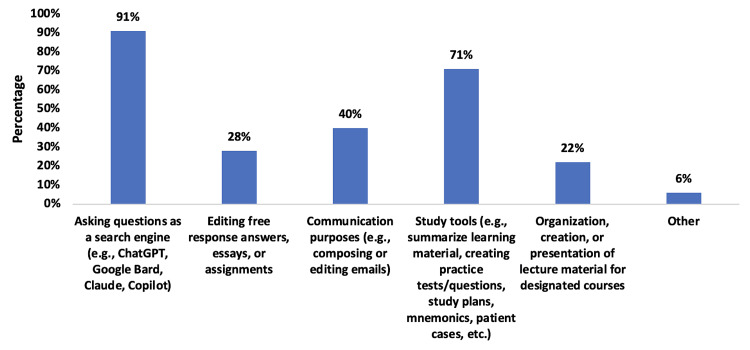
Student use of AI the week prior to beginning their clinical clerkships. Data are from survey of M2 (rising M3) students in the Transition to Clinical Clerkship Course offered in February 2025 for the second cohort of students (n=70) AI: artificial intelligence

Of the voluntary survey participants, 20 (24%) of the cohort had used AI in Spring 2024. Usage of AI in this cohort rose to 62 (77%) by the week before they began clerkships in March 2025. How these students were using AI just before the start of their clinical clerkships is displayed in Figure 2.

Asking questions as a search engine was the most common reported use (59, 91%), followed by study tools (e.g., summarizing learning material, creating practice tests/questions, study plans, mnemonics, patient cases: 46 (71%)) and communication purposes (e.g., composing or editing emails: 26 (40%)). Most students reported integrating AI once or twice a week (21, 30%) or five or more times a week (19, 27%). These results are consistent with how this cohort initially responded in Spring 2024 (data not shown).

Due to the rapidly advancing nature of AI, students found it difficult to keep up with current and new applications. As shown in Table 2, when asked to rate the relative importance of using AI, students ranked the following tasks as most important: (1) to summarize learning materials provided by instructors, (2) to summarize assigned reading material, and (3) to test understanding of what I studied.

**Table 2:** Relative importance of AI to student learning. Students were asked to rate the relative importance of each learning item as not at all important (1), somewhat important (2), important (3), or very important (4). Students' perception of the importance of AI to their learning appears stable over time as only one learning item "to develop mnemonics" showed a significant difference (p<0.05) over time AI: artificial intelligence

Learning item	May 2024 (n=20)		February 2025 (n=70)		t-statistic	P-value
	Mean	SD	Mean	SD		
To summarize learning materials provided by instructors	2.52	1.21	2.59	1.1	0.245	0.808
For help answering assigned questions	2.32	1.06	2.12	1.07	-0.769	0.447
To summarize assigned reading material	2.43	1.25	2.5	1.14	0.237	0.814
To create practice test questions or vignettes	2.09	1.14	2.28	1.09	0.697	0.49
To schedule my day's study plans	1.57	0.92	1.68	0.99	0.486	0.63
To develop mnemonics	1.64	0.83	2.26	1.14	2.769	0.008
To develop clinical cases that relate to course	1.82	0.94	1.87	0.97	0.215	0.831
To test my understanding of what I studied	2.59	1.23	2.3	1.2	-0.965	0.341
To generate flashcards	1.5	0.78	1.62	0.91	0.601	0.551
To create patient simulation encounters	1.5	0.99	1.65	0.87	0.636	0.529

Regarding career relevance (Table 3), students ranked the following as most important: (1) being able to function as a clinician without using AI modalities, (2) being able to function as a clinician using AI modalities, and (3) learning about different LLMs as they impact my profession.

**Table 3:** Students' perceptions of relevance of AI to their careers. Students were asked to rate the importance of each leaning item to their careers as not at all important (1), somewhat important (2), important (3), or very important (4). No statistically significant differences over the time points (all p>0.05) suggest stable perceptions by students of AI's career relevance AI: artificial intelligence; LLMs: large language models

Learning item	May 2024 (n=20)		February 2025 (n=70)		t-statistic	P-value
	Mean	SD	Mean	SD		
Being prepared to work using AI modalities	2.97	0.92	2.84	1	-0.638	0.526
Being able to function as clinician using AI modalities	2.83	1.02	2.97	0.93	0.598	0.552
Being able to function as clinician without using AI modalities	3.45	0.93	3.26	0.99	-0.84	0.404
Learning about different LLMs as they impact my education	2.73	1.06	2.75	1.01	0.095	0.925
Learning about different LLMs as they impact my pro	3.19	0.98	2.9	0.9	-1.411	0.163
Gaining clinical experiences using LLMs	2.65	1.15	2.74	1	0.354	0.725

**Figure 3:**
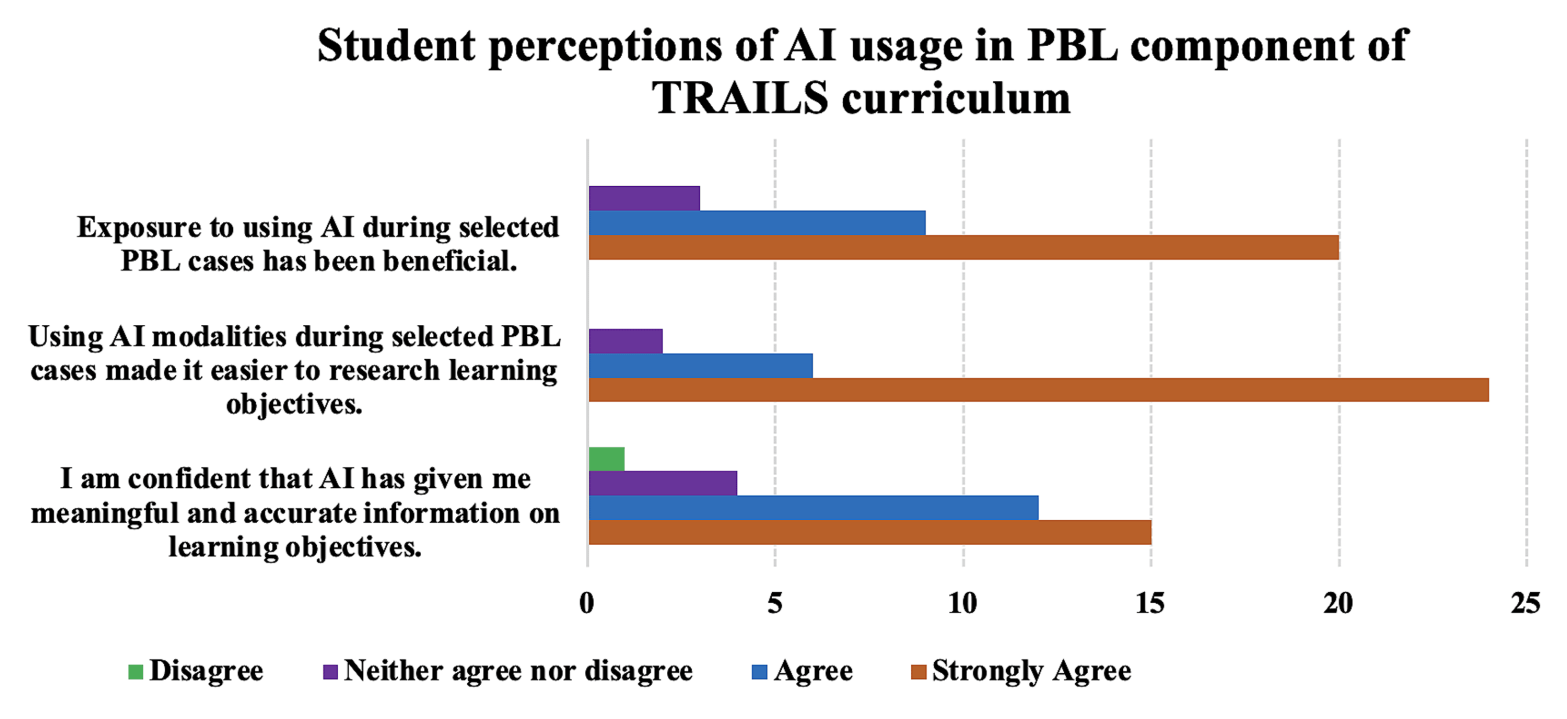
Student perceptions on AI usage during PBL. Data are from the end of course survey of M1s in December 2024 and represent a response rate of 32 students from a total of 78 potential responses AI: artificial intelligence; PBL: problem-based learning; TRAILS: Team-based Rural Applied Integrated Learning System

Student perceptions of AI usage in the PBL component of the TRAILS curriculum are shown in Figure 3.

The results indicate that the majority of M1 students who responded to the optional end-of-course survey distributed via Qualtrics found exposure to AI beneficial: 32 (strongly agree: 63%, agree: 28%, neither agree nor disagree: 6%). Similarly, most students thought that using AI made it easier to find content related to their self-selected learning objectives: 32 (strongly agree: 75%, agree: 19%, neither agree nor disagree: 6%). However, more students were unsure about AI's ability to provide meaningful and accurate information: 32 (strongly agree: 47%, agree: 38%, neither agree nor disagree: 13%, disagree: 3%).

Finally, since the data we have gathered for analysis come from student satisfaction surveys, we have compiled representative qualitative comments from our second cohort of students just before they began their clinical clerkship rotations as new M3s and from our third cohort of students as they completed their PBL activities in Fall 2024 (Table 4).

**Table 4:** Selected qualitative comments regarding AI from M2s (rising M3s) before they begin clinical clerkships and of M1s after a semester where they used AI during PBL AI: artificial intelligence; PBL: problem-based learning; SOAP: Subjective, Objective, Assessment, and Plan; LOs: learning objectives

Category	Feedback
Cautious but positive and/or optimistic outlook before beginning clerkships (M3)	It is both important to learn how to practice medicine with and without AI as we move forward into the field.
	I believe it’s important to be familiar with the advances in AI applied to health sciences and more importantly the limitations.
	I believe that it is important to start integrating AI into our education because I believe it will have a big place in the future of healthcare and we need to know how to use it effectively.
	I love it for double-checking or getting explanations about certain clinical differentials or treatments as well as in class questions.
	I think it is more of an exciting time than it is a harrowing one, and while I do think there will be some negative outcomes from the integration of AI into medicine, I am more optimistic that the positive outcomes will outweigh them.
	AI can assist in medical education by using it as an additional resource, but it should not replace learning skills.
	I think it will be great for scribing and writing SOAP notes and maybe helping create differentials. It should never replace physicians.
Cautious, negative, and/or pessimistic outlook before beginning clerkships (M3)	If we do not understand how AI is used, we will get left behind. Further, if we are not involved in the conversation, we will be left out of the conversation. History has lots of great examples of professions and industries that failed to adopt and adapt.
	Sometimes it can be wrong, and the mnemonics it gives are very terrible.
	Pretty sure using AI is prohibited by the school. Never used it before. Sorry.
	I think AI is overhyped and relied on way too much by students. I feel like it allows people to be lazy and not think critically...when people use it to craft entire papers, write emails, read papers, etc., we start to take the brain power out of learning.
	I am personally very wary of using these tools at the present due to the "hallucination" phenomenon common in AI where the tool provides confident but completely false or manufactured information and presents it as fact…I will personally not be making frequent use of AI tools and will be encouraging fellow students to be very cautious when doing so themselves.
	Concerned about health data protection.
Positive and/or optimistic outlook after completing PBL in Fall (M1)	AI made finding the LOs easier. More efficient and effective.
	Very useful in organizing the massive amount of information that we are expected to learn on a daily basis and a great place to start when it comes to researching LOs. I enjoyed it greatly and it aided in my learning.
	Allowing us to do our research with and without AI helped compare each option and demonstrated the multitude of uses for AI in our learning.
	I strongly believe that AI is the way of the future in clinical and diagnostic medicine.
Negative and/or pessimistic outlook after completing PBL in Fall (M1)	I'm not convinced that the understanding of the material was improved at all. It's possible that the material was understood more superficially because of the AI assistance.
	Sometimes it was difficult to understand how accurately a topic was explained by AI when I hadn't learned it yet myself. ... I also think it robs us of the value that comes with the effort that goes into research. Effortful learning is effective learning. AI tends to remove some of that effort.
	It's easier to give the impression of understanding material, but there is a danger of having a more superficial understanding if the student relies on AI assistance too readily.
	I worry about using AI without double-checking it. I don't trust that it knows what it is talking about sometimes, as it may have been trained on incorrect information.
	I fear that doctors may lack on their knowledge base if they can get the answer to any question at the touch of a button in the future.

We have grouped the comments as positive with an optimistic outlook for the use of AI and negative with a pessimistic outlook for the use of AI.

## Discussion

Generative AI encompasses the creation of machines or systems that can perform tasks typically requiring human intelligence. The development of LLMs has pushed boundaries in tasks previously limited to humans [6-10], leading to new policies emphasizing responsible integration, ethical considerations, and equitable access to AI in medical education [4]. Like many other institutions [11], we are rapidly adapting our curriculum by first evaluating the prevalence and utilization of AI modalities during the pre-clerkship phase. Our pre-clerkship phase of the TRAILS curriculum lasts 17 months, with students taking the United States Medical Licensing Examination (USMLE) Step 1 shortly after completion. Following a brief Transition to Clinical Medicine Course (less than three weeks), students begin clerkships in March of their second year. The increased usage of LLMs over the past two years has raised questions about their safe integration into our compressed curriculum. Informal conversations revealed that many students were already using AI to generate questions and summarize handouts. National satisfaction instruments such as the Post-MCAT Questionnaire (PMQ) [12], Matriculating Student Questionnaire (MSQ) [13], and Year Two Questionnaire (Y2Q) [14] do not yet capture student usage of AI modalities. Our students, like others nationally, use Anki and similar methods for studying, and the national trend shows declining use of commercial MCAT practice tests while free online flashcard generators have increased [12]. This trend is significant as AI flashcard generators are now freely available.

Subscription costs for premium AI platforms can hinder equitable student access. While the University of California System recently announced free ChatGPT Plus access for all students, we could only fund M1 students starting in Fall 2024. Despite formally incorporating AI into the PBL component of TRAILS for M1s, few students initially applied for reimbursement. This suggests potential concerns about AI use, lack of guidance from upper-class students, or uncertainty about its appropriate usage. This may also be reflected in the end-of-course survey results, with only 32 of 78 M1 students responding (a 41% response rate). This may indicate a self-selection bias, where only students enthusiastic about AI responded, potentially leading us to overestimate student interest in using AI as they progress through the preclinical curriculum. Additionally, we recognize that many students tend to "tune out" survey requests by the end of their first semester unless they are part of formal course evaluations. Our end-of-course survey was voluntary.

Our initial survey in Spring 2024 also had limited participation, likely due to the voluntary nature of the survey and lack of clarity in the school's policies related to AI usage. A schoolwide email about AI use lacked specificity about permissible uses, and this lack of clarity is likely reflected in recent survey comments. Based on surveying students on AI usage, we found that medical students who began in Fall 2023 were overall more familiar with AI modalities at the completion of their first academic year (Spring 2024) compared to the newer entering class in Fall 2024 at matriculation. Among survey participants who reported using AI tools (19, 21.8%) at the completion of their first year, ChatGPT (free and subscription-based) was the most popular tool. When surveyed again during the last week before starting clerkships, the majority (70, 89%) reported using AI tools. ChatGPT's free version remained the most popular tool (60, 88%). It's important to note this cohort did not have access to the grant funds provided to the later cohort, which began in Fall 2024.

AI policies are evolving rapidly worldwide. In February 2025, the European Union implemented stricter policies on safe AI use that will likely impact medical education globally [15]. Although we are not aware of our students using Manus and DeepSeek, as of March 11, those platforms have been officially banned in our university and by the State of Tennessee in order to mitigate significant data privacy and cybersecurity concerns. Throughout 2024, many educational and healthcare organizations grappled with developing ethical guardrails for AI use. This includes the creation of an International Advisory Committee for Artificial Intelligence (IACAI), which has developed vision statements addressing AI's role in medical education, focusing on culture and integration, literacy, ethics, and more [16]. Additionally, there are recommendations for AI in the biological and biomedical sciences, creating a dynamic regulatory environment [17]. Since our institution is developing new policies to keep pace with evolving medical education, some students may have avoided participating in our surveys due to unclear guidelines and fear of repercussions. For example, an Emory University student was suspended in 2023 for developing and using Eightball, an AI tool [18]. It's essential to reassess each matriculating class's understanding of AI in medical education.

Survey data from February 2025, just before our second cohort of students in TRAILS began clerkships, showed that 89% had been exposed to AI in controlled settings after our AI-related activities had been in place for at least one semester. While we did not require AI use, we encouraged it through PBL and TBL. Given the rapid pace of AI adoption, our initial findings suggest that students are already integrating these tools into their learning. Based on the satisfaction of both students and faculty facilitators with AI in controlled PBL settings during Fall 2024 for our third cohort, we now encourage its use throughout the PBL components of the curriculum. As students move on from the preclinical to clinical phases of our curriculum, we advise them to be enthusiastic but cautious about using AI. Notably, 16% of survey respondents expressed uncertainty about the accuracy of AI-generated information in the controlled PBL setting. This healthy skepticism is appropriate as students navigate the rapidly evolving landscape of AI in medicine. Qualitative student comments appropriately reflect this skepticism.

In Spring 2024, faculty were invited to participate in our initial survey; however, responses from faculty were lower than expected and thus not included herein. We attribute low faculty responses to uncertainties about institutional guidelines and a general lack of familiarity with AI. To address this, our institution developed a workshop in Summer 2024 to introduce AI to faculty. We now encourage attendance at AAMC and International Association of Medical Science Educators (IAMSE) webinars on this topic. While information is anecdotal at present, many of our faculty are using AI to modify and develop new teaching materials. For example, our PBL facilitators routinely refer students to use sites like OpenEvidence when questions arise during group discussions. By sharing our experience, we aim to help other institutions evaluate their own AI integration approaches and gain insights into stakeholder perceptions regarding implementation.

Study limitations

The voluntary nature of our surveys resulted in low response rates, potentially creating self-selection bias where only students enthusiastic about AI participated, leading to overestimation of student interest. Further, the limited sample size from a single institution (Quillen College of Medicine) restricts the generalizability of our findings to other medical schools with different curricula, student populations, or institutional resources. Faculty perspectives were largely excluded due to poor survey response rates, leaving a significant gap in understanding stakeholder views on AI integration within our curriculum. Since courses in our curriculum are graded as Pass/Fail, the study does not report on educational outcomes such as grades or test scores. The unequal sample sizes between survey time points (n=20 in May 2024 vs. n=70 in February 2025) may have affected the statistical power of our comparisons. Additionally, the rapid evolution of AI technology and policies during the study period, combined with unclear institutional guidelines early in implementation, may have influenced student participation and responses, potentially affecting the validity of the findings.

## Conclusions

This study demonstrates that medical schools can successfully implement a structured approach to integrate generative AI into their preclinical curricula while maintaining academic integrity and ethical standards. The key to successful AI integration lies in establishing clear professionalism guidelines that define appropriate versus inappropriate uses, ensuring equitable access to AI platforms, introducing AI tools in controlled educational settings with structured evaluation criteria, and providing specialized training on ethical AI use as students transition to their clinical training. The approach emphasizes the importance of preparing future physicians to function effectively both with and without AI assistance, fostering healthy skepticism about AI-generated information while recognizing its potential benefits for learning and clinical practice. This balanced integration strategy provides a practical framework that other medical schools can adapt to their own curricula, addressing the urgent need for responsible AI adoption in medical education as these technologies become increasingly prevalent in healthcare settings.

## Appendices

**Table 5:** Survey items 1-10 AI: artificial intelligence; LLM: large language model Credits: Gabrielle Rueff, Paul Monaco, Antonio E. Rusinol, and Mark Hernandez. Modification of the survey has been published under a Creative Commons License [5]. The survey was administered via Qualtrics and approved by the East Tennessee State University (ETSU) Institutional Review Board (IRB) (approval number: 0524.7e-ETSU)

1. Position at ETSU					Response (check one)	
Clinical Science Faculty						
Basic Science Faculty						
Other Faculty						
Pre-clerkship Student						
Clerkship Student						
Staff Member						
2. Do you use generative AI for learning/professional purposes at ETSU?					Response (check one)	
Yes, I have used generative AI tools						
No, I have never used generative AI tools						
No, I have heard of it, but have never used AI tools						
No, I have never used generative AI tools, but I am planning on using them						
No, I am unaware of what generative AI tools are						
3. If you answered YES to item 2, select all that apply					Response (check all that apply)	
Asking questions of a search engine (e.g., ChatGPT, Google Bard, Claude, Copilot)						
Editing free response answers, essays, or assignments						
Communication purposes (e.g., composing or editing emails)						
Study tools (e.g., summarizing learning material, creating practice tests/questions, study plans, mnemonics, patient cases, etc.)						
Organization, creation, or presentation of lecture material for designated courses						
Other						
4. How often did you integrate AI modalities for learning/professional purposes during the last month?					Response (check one)	
Five or more times a week						
Once or twice a week						
Once a week						
Once a month						
A few times in the past month						
Did not use any generative AI tools in the past month						
5. Are you a student currently enrolled at ETSU?					Response (check one)	
Yes, I am currently enrolled at ETSU						
No, I am not enrolled at ETSU						
I am a faculty member currently enrolled in courses at ETSU						
6. Which college are you enrolled in at ETSU?					Response (check one)	
College of Medicine						
College of Pharmacy						
College of Nursing						
College of Allied Health						
College of Rehabilitative Sciences						
College of Social Work						
Prefer not to answer						
7. If you are a student and answered YES for use of AI for study tools, then please rate the relative importance of each item as it relates to your learning of the health sciences	Not at all important	Somewhat important		Important		Very important
To summarize learning materials provided by instructors						
To summarize assigned reading material						
To create practice test questions or vignettes						
To schedule my day's study plans						
To develop mnemonics						
To develop clinical cases that relate to the course						
To test my understanding of what I studied						
To generate flashcards						
To create patient simulation encounters						
For help answering assigned questions						
8. An LLM is a type of AI program that can recognize and generate text, among other tasks. LLMs are trained on huge sets of data. LLMs are built on machine learning, specifically, a type of neural network called a transformer model. For each of the following items below, please rate the relative importance of each item as it relates to your career in the health sciences profession.	Not at all important	Somewhat important		Important		Very important
Being prepared to work using AI modalities						
Being able to function as clinician using AI modalities						
Being able to function as clinician without using AI modalities						
Learning about different LLMs as they impact my education						
Learning about different LLMs as they impact my profession						
Gaining clinical experiences using LLMs						
9. Which generative AI tools have you used or are you familiar with?			Response (check all that apply)			
ChatGPT (free version)						
ChatGPT (subscription only)						
Microsoft Copilot						
Google Gemini						
Bard						
DALL-E 2 OpenAI						
Claude						
Audiogen						
SlidesAI.io						
Stability AI						
Adobe Firefly						
Canva AI						
Piktochart						
Infogram						
Visme						
DeepL						
Amazon Bedrock						
GitHub Copilot						
Other (please specify)						
10. Use this space to describe any additional thoughts or perceptions you may have about integrating AI modalities into health sciences professions education.						
